# In Vitro Digestibility, In Situ Degradability, Rumen Fermentation and N Metabolism of Camelina Co-Products for Beef Cattle Studied with a Dual Flow Continuous Culture System

**DOI:** 10.3390/ani9121079

**Published:** 2019-12-03

**Authors:** Hèctor Salas, Lorena Castillejos, Montserrat López-Suárez, Alfred Ferret

**Affiliations:** Animal Nutrition and Welfare Service (SNIBA), Department of Animal and Food Science, Universitat Autonòma de Barcelona, 08193 Bellaterra, Spain; hectorsalasolive@gmail.com (H.S.); Montserrat.Lopez.Suarez@uab.cat (M.L.-S.); Alfred.Ferret@uab.cat (A.F.)

**Keywords:** beef cattle, protein sources, camelina co-products, rumen microbial fermentation

## Abstract

**Simple Summary:**

Currently, vegetable protein sources such as soybean meal and rapeseed meal are expensive and with volatile prices. These economic circumstances are driving the research of potential new protein resources for beef cattle diets that can reduce the ration cost without compromising animal productive yields. As possible candidates, camelina meal and camelina expeller have been studied; they are co-products with a high protein percentage, obtained after oil extraction from the oil seeds of *Camelina sativa*. The objectives of this study were to characterize these camelina co-products and ascertain if they could be useful ingredients for beef cattle diets. The results indicate that the diets formulated with camelina meal and camelina expeller do not show differences in the efficiency of microbial protein synthesis compared to the current reference proteins, camelina meal diet being the most similar to soybean meal and rapeseed meal diets, and camelina expeller the diet with the highest fermentation potential. The results of soybean meal as an individual ingredient reveal more differences with camelina co-products. In vivo studies are necessary to draw conclusions, but in vitro results obtained suggest that camelina meal and camelina expeller are potential substitutes for rapeseed meal in beef cattle diets.

**Abstract:**

Camelina meal (CM) and camelina expeller (CE) were compared with soybean meal (SM) and rapeseed meal (RM). Trial 1 consisted of a modified Tilley and Terry in vitro technique. Trial 2 was an in situ technique performed by incubating nylon bags within cannulated cows. Trial 3 consisted in dual-flow continuous culture fermenters. In Trial 1, CM, CE and RM showed similar DM digestibility and OM digestibility, and SM was the most digestible ingredient (*p* < 0.05). Trial 2 showed that CE had the numerically highest DM degradability, but CP degradability was similar to RM. Camelina meal had a DM degradability similar to SM and RM and had an intermediate coefficient of CP degradability. In Trial 3, CE diet tended to present a higher true OM digestibility than SM diet (*p* = 0.06). Total volatile fatty acids (VFA) was higher in CE and CM diets than in SM diet (*p* = 0.009). Crude protein degradation tended to be higher (*p* = 0.07), and dietary nitrogen flow tended to be lower (*p* = 0.06) in CE diet than in CM diet. The efficiency of microbial protein synthesis was not affected by treatment (*p* > 0.05). In conclusion, CE and CM as protein sources differ in CP coefficient of degradability but their results were similar to RM. More differences were detected with regard to SM.

## 1. Introduction

Meeting protein requirements in ruminant nutrition can be costly. The main reasons are the high and unstable prices of protein sources, such as soybean meal, and their availability, which is affected by global trade [1]. This situation makes it necessary to search for new alternatives to replace totally or partially the protein sources currently used in ruminant diets. In this line of research, *Camelina sativa* co-products could be one such alternative. *Camelina sativa* or false flax is an oilseed crop of the Brassica family, which originates from the Mediterranean and Central Asia. It is an annual or overwintering herb with low agronomic requirements [2] and is more tolerant to frost, heat, and drought than other plants of the same family [3], such as rapeseed meal. The biofuel industry’s growing interest in its cultivation is attributable to the 40% oil content of the seed, which is used to produce biodiesel [4,5,6,7,8]. When oil is extracted from the seed, camelina expeller (CE) and camelina meal (CM) are produced, the former being obtained after mechanical oil extraction of the seed and the latter after mechanical and subsequent chemical oil extraction. According to Zubr [9], the resulting meal after oil extraction contains 30–40% CP and 12% fiber. However, the main limitation of using CM and CE is related to the presence of anti-nutritional components. Camelina co-products contain glucosinolates and erucic acid [10], which, according to Tripathi and Mishra [11] affect the thyroid and the cardiovascular system. That said, CM has been used in beef steers without effect on growth performance or thyroid function [12,13]. Our hypothesis was that given the high protein content of both co-products [14], CE and CM could be alternative protein sources in ruminant nutrition. The aims of this study were to characterize these camelina co-products and to compare in vitro beef cattle diets made either with them or with more commonly used protein sources like soybean meal and 00-rapeseed meal.

## 2. Materials and Methods

Animal procedures were approved by the Institutional Animal Care and Use Committee (reference CEEAH 1585) of the Universitat Autònoma de Barcelona (Spain), in accordance with the European directive 2010/63/EU.

Three different trials were conducted to achieve the objectives. Trial 1 was performed to determine the in vitro digestibility of four protein ingredients commonly used in cattle diets (Table 1). Two of them were sources supplied by the feed industry and frequently used for beef cattle: Soybean meal 44% CP (SM) and 00-rapeseed meal (RM), while the other two were CE and CM, supplied by Camelina Company, S.L. (Madrid, Spain). The CE and CM samples were selected according to their chemical composition similarity to others studies in the case of CE [8,15] and to RM in the case of CM. To select the SBM and RM samples, we performed chemical analyses of different SBM and RM varieties and selected those most similar to the most commonly used varieties in beef diets in our commercial conditions. In Trial 2, the in situ technique was used to estimate the dry matter (DM) and the crude protein (CP) effective degradability of these protein ingredients. Finally, in Trial 3 a dual-flow continuous culture system was used to study the true digestibility, rumen fermentation and nitrogen metabolism of four isoenergetic and isonitrogenous diets (Table 2). Each one was formulated with one of these protein sources and for a targeted gain of 1.4 kg/day according to Fundación Española para el Desarrollo Animal (FEDNA) [16] recommendations for beef cattle. All diets were designed with a 90:10 concentrate to barley straw ratio.

### 2.1. Chemical Composition

Chemical composition of protein sources used in Trial 1 and Trial 2, and composition of ingredients and chemical composition of diets in Trial 3 are described in Table 1 and Table 2, respectively. Samples, of either ingredients or diets, were ground to pass through a 1-mm sieve and analyzed in duplicate. The dry matter (DM) content was determined by drying samples for 24 h in a 103 °C forced air oven, and organic matter (OM) was determined after ignition of a sample in a muffle furnace at 550 °C overnight according to AOAC ID 950.05 [17]. Nitrogen content was determined by the Kjeldahl procedure AOAC ID 976.05 [17]. Ether extract (EE) was performed according to AOAC ID 920.30 [17]. The neutral detergent fiber (NDF) and acid detergent fiber (ADF) contents were determined sequentially by using an Ankom Fiber Analyzer (Ankom Technology, Fairport, NY, USA) in accordance with the methodology provided by the company. This is based on the procedure of Van Soest et al. [18] using a thermostable α–amylase and sodium sulfite. Ash determination was performed at the end of the sequential process to express fiber results on an ash-free basis. In the case of both camelina co-products, the concentration of the α–amylase and sodium sulfite were doubled compared to the original procedure to achieve acceptable repeatability of the determination. The lignin content was determined after fiber procedures using sulfuric acid 72%. Neutral detergent insoluble crude protein (NDICP) and acid detergent insoluble crude protein (ADICP) were determined by the aforementioned Kjeldahl procedure in residues obtained after fiber determinations. Gross energy content was measured by completely burning a sample of the ingredients to measure the heat produced using a bomb calorimeter (Parr 6300, Parr Instrument Company, Moline, IL, USA). The allyl isothiocyanate level was determined by a destilation-volumetry procedure according to the European Directive 71/250/EEC. The erucic acid content was analyzed by chromatography (Model 6890, Hewlett Packard, Palo Alto, CA, USA), according to American Oil Chemists’ society (AOCS) method CE 2-66 [19].

### 2.2. Trial 1: In vitro Digestibility

In vitro DM and OM digestibility of samples were obtained following the Tilley and Terry [20] procedure modified by Stern and Endres [21]. The experiment was performed in two periods with three replicates per period. The rumen liquid obtained from two different cows fed a 60:40 forage to concentrate ratio diet was diluted with the buffer solution proposed by McDougall [22] at a ratio of 1:4 (rumen fluid:buffer solution). A sample of 0.5 g was incubated in each tube with 50 mL of mixed solution in continuous agitation and anaerobiosis, which was achieved with the introduction of CO_2_ at 39 °C. After 48 h of incubation, 0.2 g of pepsin and 2 mL of HCl were added in each tube. Tubes were maintained in continuous agitation at 39 °C for 24 h. The content of each tube was filtered using filtration crucibles, and the DM and OM of the residue obtained were determined as mentioned previously.

### 2.3. Trial 2: In Situ Degradability

Two cannulated cows fed ad libitum with a 60:40 forage to concentrate ratio diet were used. The DM and CP in situ degradability analysis was carried out by incubating nylon bags (Ankom Technology Corporation, Fairport, NY, USA) in the rumen, which were 5 cm × 10 cm with a pore size of 50 µm containing 1 g of sample that was ground to pass through a 1.5-mm sieve. In three repeated periods, one bag per sample, animal and hour was inserted into the rumen at 8.30 am directly before feeding. Incubation periods were 2, 4, 6, 8, 12, 24, 48 and 72 h. After rumen incubation, bags were subjected to a washing procedure consisting of three washing cycles of 5 min, and immediately frozen at −18 °C for further analyses. The 0-h nylon bags were treated with the same methodology but without passing through the rumen. Degradation of DM and CP parameters was calculated using the equation of Ørskov and McDonald [23]:

D = *a* + *b* (1 − e ^−ct^) (1)

where D is the disappearance of either DM or CP at time t; *a* is an intercept representing the DM or CP soluble fraction, *b* is the fraction of insoluble but degradable DM or CP, *c* is the rate of disappearance of fraction *b*, and t is the time of incubation. The non-linear parameters *a*, *b* and *c* were estimated by an iterative least-squares procedure of SAS (v. 9.1; SAS Inst. Inc., Cary, NC, USA). The effective degradability (ED) of DM and CP were calculated using the equation:

ED = *a* + [*bc*/(*c* + *k*)] (2)

where *a*, *b* and *c* are the same parameters as described earlier and k is the estimated rate of passage of 0.06/h according to Institut National de la Recherche Agronomique (INRA) [24].

### 2.4. Trial 3: Dual-Flow Continuous Culture System

#### 2.4.1. Fermenters

Eight 1320 mL dual-flow continuous culture fermenters developed by Hoover et al. [25] were used in two replicated periods. Each experimental period consisted of 5 days for adaptation and 3 days for sampling. Fermenters were inoculated with ruminal liquid from two dairy cows fed a 60:40 forage to concentrate ratio diet. Fermentation conditions were maintained constant with a temperature of 39 °C, and pH at 6.1 ± 0.05 by infusion of 3 N HCl or 5 N NaOH, monitored and controlled by a computer. Liquid and solid constant dilution rates (0.1 and 0.05 h-1, respectively) were obtained by a continuous infusion of artificial saliva [26]. Finally, anaerobic conditions were maintained by infusion of N2 gas at a rate of 40 mL/min. Fermenters were daily fed 90 g of diet (DM basis) in three equidistant doses of 30 g.

#### 2.4.2. Sample Collection

On the sampling days, collection vessels were maintained at 4 °C to prevent microbial activity. Solid and liquid effluents were mixed and homogenized for 1 min, and a 600 mL sample was removed by aspiration. Upon completion of each period, effluents from each fermenter collected over the three sampling days were composited and mixed and homogenized for 1 min. Subsamples were taken for total N, ammonia N, and VFA analyses. The remainder of the sample was lyophilized. Dry samples were analyzed for DM, ash, NDF, ADF, and purine contents.

Bacteria were isolated from the fermenter contents on the last day of each experimental period. Solid and liquid associated bacteria were isolated using a combination of several detachment procedures, which were selected to obtain the maximum detachment without affecting cell integrity [27]. To remove attached bacteria, 100 mL of a 2 g/L methylcellulose solution and small marbles (thirty measuring 2 mm in diameter, and fifteen of 4 mm) were added to each fermenter, incubated in the same fermenter contents at 39 °C, and mixed for 1 h. After incubation, fermenter contents were refrigerated for 24 h at 4 °C, and subsequently agitated for 1 h to dislodge loosely attached bacteria. Finally, the fermenter contents were filtered through cheesecloth and washed with saline solution (8.5 g/L NaCl). Bacterial cells were isolated within 4 h by differential centrifugation at 1000× *g* for 10 min, to obtain a supernatant without feed particles, which was then centrifuged at 20,000× *g* for 20 min to isolate bacterial cells. Pellets were rinsed twice with saline solution and recentrifuged at 20,000× *g* for 20 min. The final pellet was recovered with distilled water to prevent contamination of bacteria with ash. Bacterial cells were lyophilized and analyzed for DM, ash, N, and purine contents. Digestion of DM, OM, NDF, ADF and CP, and flows of total, non-ammonia, microbial, and dietary N were calculated as described by Stern and Hoover [28].

#### 2.4.3. Chemical Analyses

Effluent DM was determined by lyophilizing 200 mL aliquots in triplicate. The DM, OM, total N, NDF, ADF, lignin and EE content of the lyophilized effluents, bacterial samples and diets were determined as described in Section 2.1. Effluent ammonia N was analyzed by colorimetry as described by Chaney and Marbach [29], where 4 mL of a 0.2 N HCl solution were added to 4 mL of filtered rumen fluid and frozen at −20 °C until later analysis. Samples were centrifuged at 15,000× *g* for 15 min, and the supernatant was used to determine ammonia N. Effluent samples for VFA determination were prepared as described by Jouany [30] and analyzed by gas chromatography: 1 mL of a solution made up of a 2 g/L solution of mercuric chloride, 0.002 mg/L of 4-methylvaleric acid as an internal standard, and 2 g/L orthophosphoric acid was added to 4 mL of filtered rumen fluid and frozen at −20 °C until later analysis. Samples were centrifuged at 3000× *g* for 30 min, and the supernatant analyzed by gas chromatography (Model 6890, Hewlett Packard, Palo Alto, CA, USA) using a polyethylene glycol TPA treated capillary column (BP21, SGE, Europe Ltd., Buckinghamshire, UK). The dimensions of the column were 30 m × 0.25 mm ID, with 0.25 µm film thickness. The injector was set at 275 °C with a split ratio of 4:1. Helium was used as the carrier gas. The initial temperature was 85 °C for 1 min, and increased by 3C/min until a final temperature of 220 °C was reached, and held for a further 2 min. The detector temperature was 275 °C. Samples of lyophilized effluent and bacterial cells were analyzed for adenine and guanine content by HPLC as described by Balcells et al. [31], using allopurinol as internal standard.

### 2.5. Statistical Analysis

Data from the in vitro digestibility experiment were analyzed using the MIXED procedure of SAS (v. 9.1; SAS Inst. Inc., Cary, NC, USA). The model contained the fixed effect of treatment, and the random effect of period. Statistical analysis of the dual-flow continuous culture system data was performed using the MIXED procedure, and multiple comparisons were performed by LSMEANS adjusted with the Tukey test using SAS (v. 9.1; SAS Inst. Inc., Cary, NC, USA). The model contained treatment as the fixed effect and period as the random effect. Statistical significance was declared at *p* < 0.05 and differences among means with 0.05 < *p* < 0.10 were considered tendencies.

## 3. Results

### 3.1. In Vitro Digestibility with the Two-Stage Tilley and Terry Technique

In vitro DM digestibility (DMD) and OM digestibility (OMD) values are presented in Table 3. Soybean meal was the protein source with the highest digestibility (*p* < 0.05), both DMD and OMD. There were no significant differences (*p* > 0.05) among CM, CE and RM in DMD, and OMD values.

### 3.2. In Situ Rumen Degradation

**Figure 1:**
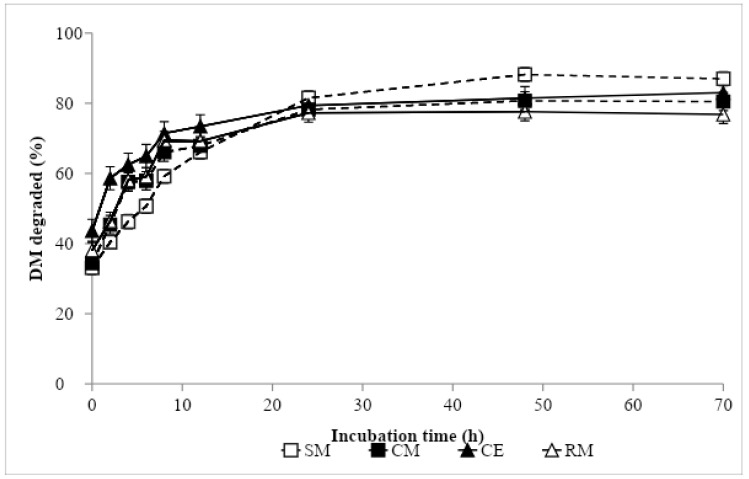
In situ dry matter degradation curve of protein sources: SM, Soybean meal 44% CP; CM, camelina meal; CE, camelina expeller; RM, rapeseed meal 00.

**Figure 2:**
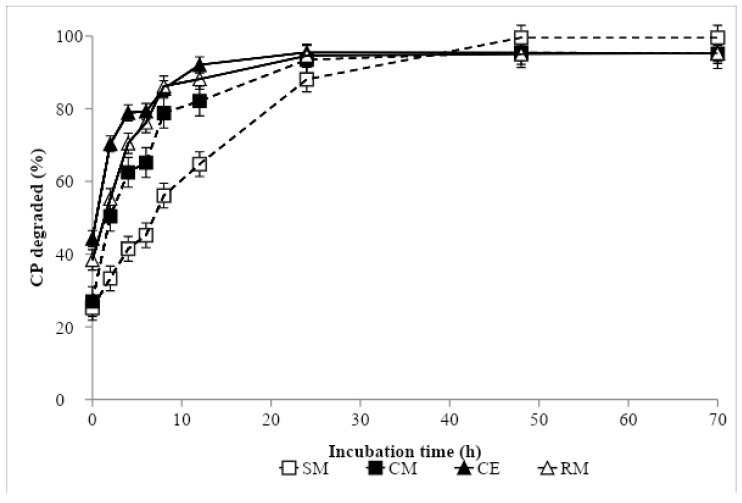
In situ crude protein degradation curve of protein sources: SM, Soybean meal 44% CP; CM, camelina meal; CE, camelina expeller; RM, rapeseed meal 00.

Nonlinear parameter estimates and ED values are presented in Table 4, and ruminal degradation kinetics of DM and CP are shown in Figure 1 and Figure 2, respectively. The greatest DM soluble fraction was recorded with CE, while the remaining protein sources showed similar values. In contrast, the greatest fraction *b* was obtained in SM, being intermediate in CM and RM, and lowest in CE. The DM rate of the disappearance of fraction *b* was similar in CM and CE, and in an intermediate position between SM, with the lowest value, and RM, with the greatest. The ED of DM was similar in SM, CM and RM, and the greatest value was recorded in CE. In descending order, the CP *a* fraction values came out as follows: CE, RM, CM and SM. Soybean meal showed the highest *b* value, followed by CM, RM and CE. The lowest rate of CP degradation was found in SM, followed by CM and RM, and the greatest was recorded in CE. The lowest ED of CP was obtained in SM, the greatest in CE and RM, and the value was intermediate for CM.

### 3.3. Dual-Flow Continuous Culture System

Results of dual-flow continuous culture system are presented in Table 5, Table 6 and Table 7.

Apparent digestibility of DM, NDF and ADF, and true DM digestibility were not different among diets (Table 5). In contrast, true OM digestibility tended to be affected by diet, with the highest value in CED and the lowest in SMD. Total VFA was higher in CED and CMD than in SMD (*p* = 0.009), but there was no difference between these diets and RMD (Table 6). Butyrate proportion was lower in CMD than in SMD (*p* = 0.016), but the proportion detected in CED and RMD was not different from that detected in CMD and SMD. There were no differences among diets in acetate, propionate, iso-butyrate, valerate, and iso-valerate proportions, the BCVFA, and the acetate to propionate ratio. Table 7 shows the results on nitrogen metabolism. There were no diet effects on total NH3-N, and flows of total, ammonia, non-ammonia and bacterial N, and EMPS. However, dietary N flow tended to be lower in CED than in CMD (*p* = 0.06), and CP degradation tended to be higher in CED than in CMD (*p* = 0.07).

## 4. Discussion

Chemical composition of feedstuffs commonly used in beef cattle diets, like SM and RM, was in accordance with published values [24,32]. Comparing both *Camelina sativa* co-products, CM contained higher values of CP, NDF, ADF and lignin compared to CE, but lower EE content. The CP content of the CM used in the present study was similar to the values referenced in the literature [8,33]. On the contrary, the EE content of these references was closer to the value found in CE. In reference to the anti-nutritional factors, the content of allyl isothiocyanate, as a major metabolite of glucosinolates of CM and CE, was below that of RM, an ingredient that is considered to have a negligible amount of glucosinolates. Tripathi and Mishra [11] obtained values between 0.3 mg/g and 2.1 mg/g of allyl isothiocyanate in different varieties of RM obtained in diverse oil extraction processes. Values of erucic acid presented by CM and CE were below 1% of the fat fraction that is considered to be the threshold of a rapeseed meal zero erucic acid variety [16]. Therefore, the comparison between camelina co-products and RM suggests that their use would not represent a nutritional problem for beef cattle nutrition.

In vitro DM and OM digestibility were not different in both camelina co-products. These digestibility values were similar to those recorded with RM but lower than in SM. The higher CP content and the lower fiber content of SM would explain these higher digestibility coefficients. Yong-Gang Liu et al. [33] and Moss and Givens [34], also using in vitro studies, reported equivalent OMD coefficients for SM and RM (0.89 and 0.74, respectively) to those reported in the present study.

Effective DM degradation was higher in CE than in CM. This difference would be related with the *a* and *b* fractions, which were higher and lower, respectively, in CE than in CM, but without differences in the rate of DM disappearance. Dry matter ED of CM was close to the values recorded in RM and SM. However, this result was obtained with different kinetic parameters because although the *a* fraction was similar among these protein sources, the b fraction was higher in SM and lower in CE, while rate of DM disappearance was higher in RM and lower in SM. In the comparison between SM and RM, our results agree with Heendeniya et al. [35] and Wulf and Südekum [36], who presented similar DM kinetic parameters between SM and RM, with a higher insoluble but degradable fraction and slower degradation rate in SM compared with RM. In contrast, Prestløkken [37] (1999) did not report differences between SM and RM, and Maxin et al. [38] found a greater DM degradation rate and ED in SM than in RM. The highest *b* fraction recorded in SM could be explained by its chemical composition: High CP content, low fiber content, and low content of NDICP and ADICP, resulting in the highest DMD and OMD in comparison with the remaining protein sources.

Effective CP degradation of CE was higher than that of CM, with a higher *a* fraction and rate of CP disappearance and a lower *b* fraction. Lawrence and Anderson [39] studied the kinetic parameters of a CM and recorded CP degradability very similar to the result obtained in the present study. However, the chemical composition of their CM was closer to our CE, because their EE content was 143 g/kg DM, similar to the 135 g/kg DM of our CE and different from the 13 g/kg DM of our CM. Camelina expeller and RM showed the same ED of CP, but the *a* fraction and rate of disappearance were lower and the *b* fraction higher in RM than in CE. The lowest ED of CP was found in SM, in comparison with the remaining protein sources. The values obtained with SM were in accordance with the literature [36,37,40]. Conversely, RM soluble fraction was very high in comparison with Prestløkken [37], Wulf and Südekum [36] and Heendeniya et al. [35], and close to the result obtained by Maxin et al. [38].

Chemical composition of the diets tested in the dual-flow continuous culture system confirmed that they were formulated to be isonergetic and isonitrogenous, with only a slight decrease in the CP content in the SMD diet. No differences among diets in apparent digestibilities and true DMD were observed. In contrast, Brandao et al. [41], in a dual-flow continuous culture system trial, observed lower NDF digestibility in diets with either 50% or 100% of CE instead of RM, meaning that RM was included at 10.3% and 0% (on DM basis), respectively, compared to the control diet with RM, in which RM was included at 20.6% as the main protein source. The lack of differences in our trial between SMD and RMD digestibilities is in accordance with Paula et al. [42] who also compared, in a dual-flow continuous culture system, a SM diet with RM diets with different rumen-undegradable protein (RUP) content. However, true OMD tended to be affected by diet. The trend detected in the highest true OMD value in CED compared to SMD, although not statistically different from CMD and RMD, could be related with the highest fermentation activity observed in this diet, where high values of total VFA were recorded in comparison with SMD. With similar true OMD to SMD but also CED, CMD and RMD did not differ from CED in total VFA concentration. While there were no differences among diets in acetate and propionate proportion, butyrate proportion in CMD was lower than in SMD. During the synthesis of acetate and butyrate, there is also a methane generation that reduces its energy efficiency compared with the synthesis of propionate, in the formation of which there is no loss of any carbon during the reaction [43]. Therefore, it seems that CMD could be more efficient in energy usage. This could be related with the higher total VFA concentration detected in CMD and CED than in SMD. Although there was no different BCVFA content among diets, the numerically higher value found in CED diet could be related with the high CP degradability recorded in this diet, because BCVFA are produced when rumen microbes degrade protein [44]. The highest CP degradation detected in the CED would coincide with the highest CP degradation recorded in the in situ trial. In contrast, the same did not occur in the case of SMD and RMD when comparing in situ and fermenter results. In addition we obtained a lower CP degradation in CMD than in CED, and the same result between SMD and RMD. In the in vitro experiment of Brandao et al. [41], there were also no differences between RMD and CED treatments. In the in situ trial, in accordance with Huhtanen et al. [45] and most feed tables [24,46], a greater RUP content for SM compared with RM was recorded. However, Brito et al. [47] and Brito and Broderick [48] reported no differences in omasal RUP flows when isonitrogenous RM and SM diets were compared, in accordance with our fermenter results. Higher RUP of SMD, RMD and CMD could explain the effects of diet on dietary N flow, in which a tendency was found for the lowest flow in CED, highest in CMD, and intermediate in SMD and RMD diets. This difference in the RUP content between CED and CMD could be due to the second oil-extraction chemical treatment performed in CM, which is not applied in CE. This second extraction could modify the rumen digestibility of protein by decreasing the accessibility of rumen microorganisms to protein. Chemical and physical extractions are strategies commonly used to reduce ruminal CP degradability and increase RUP [45,49]. Moreover, this could also explain the numerical results observed in bacterial N flows, which were numerically higher in CED than in the remaining diets. When comparing a high RUP diet with a basal diet, Ipharraguerre and Clark [50] reported that a significant decrease in the flow of microbial N to the small intestine occurred with RUP supplements. Hoover and Stokes [51] concluded that when rumen degradable protein is replaced by RUP, the microbial growth in the rumen can decrease. The high OMD, the high CP degradation and the low RUP of CED could have promoted the microbial growth. This would be in agreement with Santos et al. [49], who concluded that high RUP diets resulted in decreased microbial protein synthesis. However, there was no significant difference between treatments in EMPS. It is important to remark that CED and CMD presented equivalent results in EMPS than diets formulated with SM and RM, the most common protein sources used in beef cattle. The lack of differences between treatments could be related with the low NH3-N concentration recorded in all effluents, which did not attain the 5 mg/dL, concentration usually recommended to ensure maximum microbial growth [52]. However, Russell et al. [53] reported no difference in microbial growth when NH3-N concentration was below 5 mg/dL or greater than 16 mg/dL. Owens and Bergen [54] concluded that the minimum amount of NH3-N to maximize bacterial growth was 2.5 mg/dL. Other studies have also reported NH3-N concentrations below 5 mg/ dL without reporting differences in microbial yield [55]. Finally, considering that barley protein is more degradable than corn protein [56,57], the differences in CP degradation detected in the present study could also be explained by the different barley to corn ratio of the diets, this ratio being 0.90, 0.85, 0.79, and 0.72 for CED, SMD, RMD, and CMD, respectively.

The results for digestibility, rumen fermentation and nitrogen flow recorded in CMD were similar to those found in diets formulated with standard proteins such as SMD and RMD, but with an increase in total VFA concentration. These similar results could be related to the close chemical composition of the main protein sources of these diets, and especially the very similar CP digestibility detected for these diets compared with CED. As has been argued previously in the specific case of the comparison between CMD and CED, the chemical oil extraction process could decrease the protein availability for the rumen microorganisms.

## 5. Conclusions

In conclusion, although both camelina co-products showed some differences between them in CP degradability and N flows, when used as a main protein source in isocaloric and isonitrogenous diets, neither of them differed from rapeseed meal in coefficients of digestibility, rumen fermentation and nitrogen metabolism. Thus, camelina co-products could be an alternative ingredient for beef cattle diets. Further in vivo research is necessary to confirm these results and to ascertain the suitable level of inclusion of these co-products.

## Figures and Tables

**Table 1:** Chemical composition of protein sources ^1^ (DM basis).

Item	SM	CM	CE	RM
Chemical composition (g/kg)				
DM	881	915	928	883
OM	824	858	880	814
CP	467	395	351	398
EE	26.1	12.9	135.7	15.5
NDF	107	375	327	358
ADF	120	174	144	214
Lignin	5.3	40.3	25.8	88.2
NDICP	29.3	118.8	107.1	86.4
ADICP	13.5	62.7	63.0	58.2
Gross energy (Mcal/Kg)	4.38	4.35	4.96	4.29
Antinutritional factors Allyl isothiocyanate (mg/g)	-	0.09	0.09	0.13
Erucic acid (g/100g fat)	-	0.05	0.04	<0.01

^1^ SM, Soybean meal 44% CP; CM, Camelina meal; CE, Camelina expeller; RM, Rapeseed meal 00.

**Table 2:** Ingredients and chemical composition of treatment diets ^1^ tested with the dual-flow continuous culture system.

Item	SMD ^1^	CMD	CED	RMD
Ingredients (g/kg DM)				
Corn grain	376	379	343	364
Barley grain	318	272	308	286
Soybean meal 44%	114	-	-	-
Camelina meal	-	152	-	-
Camelina expeller	-	-	163	-
Rapeseed 00 meal	-	-	-	149
Barley straw	100	100	100	100
Palm oil	10	27	10	28
Soybean hulls	69	56	62	60
Calcium carbonate	9	10	10	9
Vitamin and mineral premix ^2^	4	4	4	4
Chemical composition (g/kg DM)				
DM	889	894	893	889
OM	852	860	858	854
CP	125	132	132	130
EE	35.8	43.1	43.3	45.9
NDF	217	224	242	221
ADF	112	117	124	124
Lignin	12.2	104	20.4	21.7
Gross energy (Mcal/Kg)	3.97	4.07	4.08	4.07

^1^ SMD, diet with soybean meal 44% CP; CMD, diet with camelina meal; CED, diet with camelina expeller; RMD, diet with rapeseed meal 00. ^2^ Vitamin and mineral premix: Vitamin A 8400 IU/Kg; Vitamin D3 1680 IU/Kg; Zinc oxide 85 mg/Kg; Iron carbonate 39 mg/Kg; Manganese oxide 30 mg/Kg.

**Table 3:** In vitro digestibility of protein sources ^1^.

Item	SM	CM	CE	RM	SEM ^2^	*p*-Value
Coefficients of digestibility						
Dry matter	0.74 ^a3^	0.65 ^b^	0.64 ^b^	0.62 ^b^	0.160	<0.001
Organic matter	0.87 ^a^	0.72 ^b^	0.70 ^b^	0.72 ^b^	0.131	<0.001

^1^ SM, soybean meal 44% CP; CM, camelina meal; CE, camelina expeller; RM, rapeseed meal 00. ^2^ SEM, standard error of mean. ^3^ Means with different superscript differ statistically (*p* < 0.05).

**Table 4:** In situ nonlinear estimates ^1^ and effective degradability values of dry matter (ED) and crude protein (EDCP) of protein sources ^2^.

Item	SM	CM	CE	RM
Dry matter				
*a*	0.32 ± 0.012 ^3^	0.35 ± 0.022	0.50 ± 0.013	0.37 ± 0.025
*b*	0.57 ± 0.015	0.44 ± 0.025	0.33 ± 0.015	0.40 ± 0.028
*c* (/h)	0.077 ± 0.001	0.135 ± 0.017	0.132 ± 0.014	0.180 ± 0.027
ED	0.64	0.66	0.72	0.67
Crude Protein				
*a*	0.24 ± 0.017	0.29 ± 0.027	0.47 ± 0.030	0.38 ± 0.019
*b*	0.79 ± 0.022	0.66 ± 0.031	0.48 ± 0.034	0.57 ± 0.017
*c* (/h)	0.064 ± 0.001	0.160 ± 0.017	0.249 ± 0.038	0.199 ± 0.013
ED	0.64	0.77	0.85	0.82

^1^*a*: Soluble fraction; *b*: Insoluble but degradable fraction; *c*: The rate (/h) of disappearance of *b* fraction; ^2^ SM, soybean meal 44% CP; CM, camelina meal; CE, camelina expeller; RM, rapeseed meal 00; ^3^ Mean ± standard error.

**Table 5:** Effect of treatment diet on apparent digestibility and true digestibility in the dual-flow continuous culture system.

Item	Diet ^1^				SEM ^2^	*p*-Value
	SMD	CMD	CED	RMD		
Apparent digestibility (g/kg)						
DM	442	431	413	412	34.6	0.73
OM	346	352	372	360	22.2	0.58
NDF	380	357	408	359	67.2	0.83
ADF	370	385	421	345	77.8	0.76
True digestibility (g/kg)						
DM	548	531	563	521	37.8	0.66
OM	480 ^b3^	486 ^ab^	574 ^a^	494 ^ab^	37.1	0.06

^1^ SMD, diet with soybean meal; CMD, diet with camelina meal; CED diet with camelina expeller; RMD, diet with rapeseed meal. ^2^ SEM, standard error of the mean. ^3^ Means with different superscript tended to differ (*p* < 0.10).

**Table 6:** Effect of treatment diet on volatile fatty acids (VFA) concentration and profile in the dual-flow continuous culture system.

Item	Diet ^1^				SEM ^2^	*p*-Value
	SMD	CMD	CED	RMD		
Total VFA (mM)	119.6 ^b3^	135.6 ^a^	138.8 ^a^	121.0 ^ab^	7.06	0.009
VFA (mol/100 mol)						
Acetate	45.2	46.0	46.2	44.0	2.44	0.777
Propionate	32.1	35.9	34.6	33.6	2.01	0.236
Butyrate	16.4 ^a^	12.1 ^b^	13.1 ^ab^	15.0 ^ab^	1.46	0.016
Iso-butyrate	0.39	0.41	0.45	0.43	0.04	0.357
Valerate	5.23	4.42	4.39	5.89	1.18	0.494
Iso-valerate	0.74	1.14	1.25	1.11	0.36	0.412
BCVFA ^4^ (mM)	1.14	1.55	1.70	1.54	0.40	0.399
Acetate:propionate ratio	1.42	1.31	1.35	1.32	0.12	0.773

^1^ SMD, diet with soybean meal 44% CP; CMD, diet with camelina meal; CED, diet with camelina expeller; RMD, diet with rapeseed meal 00. ^2^ SEM, standard error of mean. ^3^ Means with different superscripts differ statistically (*p* < 0.05). ^4^ BCVFA, Branch-chained VFA.

**Table 7:** The effect of treatment diet on N metabolism in the dual-flow continuous culture system.

Item	Diet ^1^				SEM ^2^	*p*-Value
	SMD	CMD	CED	RMD		
NH3-N (mg/100 mL)	1.60	1.37	1.80	2.23	0.51	0.42
N flow (g/d)						
Total	2.37	2.49	2.46	2.46	0.07	0.34
Ammonia	0.05	0.04	0.06	0.07	0.02	0.41
Non-ammonia	2.32	2.44	2.36	2.39	0.09	0.51
Bacterial	0.86	0.83	1.23	0.87	0.19	0.13
Dietary	1.46 ^ab3^	1.61 ^a^	1.13 ^b^	1.52 ^ab^	0.18	0.06
Crude protein degradation (g/kg)	229 ^ab^	196 ^b^	423 ^a^	236 ^ab^	90.62	0.07
EMPS ^4^	35.27	35.61	37.06	34.12	4.19	0.89

^1^ SMD, diet with soybean meal 44% CP; CMD, diet with camelina meal; CED, diet with camelina expeller; RMD, diet with rapeseed meal 00. ^2^ SEM, standard error of mean. ^3^ Means with different superscript tended to differ statistically (*p* < 0.10). ^4^ The efficiency of microbial protein synthesis (g bacterial N/Kg organic matter truly digested).
